# Towards a data-driven system for personalized cervical cancer risk stratification

**DOI:** 10.1038/s41598-022-16361-6

**Published:** 2022-07-15

**Authors:** Geir Severin R. E. Langberg, Jan F. Nygård, Vinay Chakravarthi Gogineni, Mari Nygård, Markus Grasmair, Valeriya Naumova

**Affiliations:** 1 Department of Research, Cancer Registry of Norway (CRN), Oslo, 0379 Norway; 2 Department of Registry Informatics, CRN, Oslo, 0379 Norway; 3 Department of Electronic Systems, Norwegian University of Science and Technology (NTNU), Trondheim, 7491 Norway; 4 Department of Mathematical Sciences, NTNU, Trondheim, 7491 Norway; 5 Machine Intelligence Department, Simula Research Laboratory, Oslo, 0164 Norway

**Keywords:** Cancer, Health care, Mathematics and computing

## Abstract

Mass-screening programs for cervical cancer prevention in the Nordic countries have been effective in reducing cancer incidence and mortality at the population level. Women who have been regularly diagnosed with normal screening exams represent a sub-population with a low risk of disease and distinctive screening strategies which avoid over-screening while identifying those with high-grade lesions are needed to improve the existing one-size-fits-all approach. Machine learning methods for more personalized cervical cancer risk estimation may be of great utility to screening programs shifting to more targeted screening. However, deriving personalized risk prediction models is challenging as effective screening has made cervical cancer rare and the exam results are strongly skewed towards normal. Moreover, changes in female lifestyle and screening habits over time can cause a non-stationary data distribution. In this paper, we treat cervical cancer risk prediction as a longitudinal forecasting problem. We define risk estimators by extending existing frameworks developed on cervical cancer screening data to incremental learning for longitudinal risk predictions and compare these estimators to machine learning methods popular in biomedical applications. As input to the prediction models, we utilize all the available data from the individual screening histories.Using data from the Cancer Registry of Norway, we find in numerical experiments that the models are strongly biased towards normal results due to imbalanced data. To identify females at risk of cancer development, we adapt an imbalanced classification strategy to non-stationary data. Using this strategy, we estimate the absolute risk from longitudinal model predictions and a hold-out set of screening data. Comparing absolute risk curves demonstrate that prediction models can closely reflect the absolute risk observed in the hold-out set. Such models have great potential for improving cervical cancer risk stratification for more personalized screening recommendations.

## Introduction

Nation-wide cervical cancer screening programs in the Nordic countries have shown to be an effective cancer prevention strategy. These programs recommend repeated screening at regular intervals to detect precancerous lesions^1^. Although the screening recommendations have become more accurate and efficient over the years, they are based on only the most recent screening results and are standardised across the whole screening population. Specifically, the *Norwegian Cervical Cancer Screening Program* (NCCSP) currently recommends a routine screening every 3 or 5 years for females aged 25–33 years and 34–69 years, provided their last screening was normal. An alternative strategy would be to adapt the recommendations to the individual risk of disease initiation as inferred from the full screening history. For instance, a more personalized approach could be to recommend a longer screening interval to a female older than 45 who had only negative results in the past, as she may be at considerably lower risk than a 30 year old female with several past abnormalities. More personalized recommendations in cervical cancer screening may reduce the large number of unnecessary screenings of females unlikely to develop the disease, while simultaneously preventing more cancer cases^2^.

A step towards more individualized recommendations is utilizing data from existing cancer screening registries to derive prediction models for the individual risk of cervical cancer development. However, the data available from these registries contain only a few variables about previous exam results, necessary to organize and run the screening programs but no information about female lifestyle or habits. Moreover, due to most females having only normal results, the distribution of results is heavily skewed towards disease-free cases, and this distribution may also be changing over time due to temporal variations in female screening and lifestyle habits. In this paper, we use data from the NCCSP to evaluate the impact of data imbalance and data drift on model performance. We adapt machine learning methods to predict the individual time-varying risk of cervical cancer and compare their performances in numerical experiments.

### Data

Our approach is based on data from the 1.7 million females in the NCCSP screening population between 1991–2015. As this data covers the Norwegian cervical cancer screening population, the prediction models derived herein can only be evaluated internally using hold-out methods. External model validation require data from a different country but differences in screening recommendations^3^ and data collection practices make it challenging to align information for comparability. However, synergy projects with Baltic countries and Sweden are being developed to investigate the potential to extending predictors to other countries.

In this paper we considered only histories with at least 3 exam results for hold-out model validation. In the NCCSP data, more than 75 % of the females have only normal results in their history. To have more variation in the training data we sampled training histories with probabilities proportional to the most severe result in each history, making it more likely to select females with at least one abnormal result. For the test set we used only histories where the first exam was taken no later than year 2000 and at the ages 20–30 (± 5 years from the recommended youngest age for the first exam). This sampling give more recent and complete test data for a comprehensive model evaluation. As our dataset ends before 2015, selecting test histories with the first exam from year 2000 gave female age range 20–53 in the test data, while the results in the training set were from female ages 20–72. The final training set included 10K histories and the test set included 50K histories.

The NCCSP data contains only the information necessary for the *Cancer Registry of Norway* to organize and run the screening program. Although previous works^4,5^ deriving prediction models for cervical cancer risk stratification leverage personal lifestyle information, this information is in general unavailable for the whole screening population. It is also typically collected only once for each female, and thus does not capture temporal variations in the data. Therefore, there is large potential and benefits in providing prediction models based on the data routinely collected by the registries, as these may be integrated directly into the cancer screening programs. Specifically, the NCCSP data consists of timestamps, three types of medical exams (*cytology*, *histology* and *human papillomavirus* (HPV)) and the corresponding results. The HPV exams were introduced around 2005 to follow up on abnormal cytology, and as our dataset ends before 2015 it contains only a few HPV results. Due to the scarcity of HPV results in our data sample, we exclude all HPV data in this study and use only cytology and histology results. However, we plan to include more recent registry data with detailed HPV information in future work.

The primary cause for cervical cancer is persistent infection with HPV. This infection may lead to the development of low-grade lesions, progressing via high-grade precursor lesions (pre-cancer) to invasive cancer^6,7^. Exposure to HPV occurs mainly via sexual contact which, together with individual lifestyle variations, makes the risk of cervical cancer both time-varying and in-homogeneous across the screening population^7^. To represent the risk of cervical cancer development, we consider three clinically actionable states, reflecting stages in disease initiation and progression. We label these states *normal*, *low-grade* and *high-grade*.

A normal state requires no additional exams before the next routine screening, while progression from normal to low-grade calls for closer follow-up – although low-grade lesions may spontaneously regress back to normal^8^. Progression from low-grade to high-grade requires immediate clinical action to prevent cancer. Each state is determined by the outcome of medical exams and corresponds to different risk-levels of disease development.

**Figure 1:**
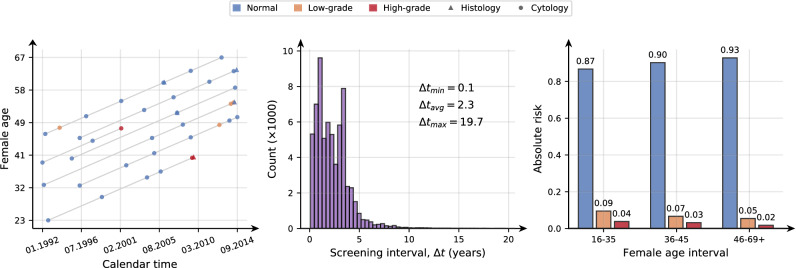
Cervical cancer screening data characteristics. Left: A Lexis diagram illustrating screening histories. Each history is depicted as a gray line spanning from the first to the last visit. Visits are indicated by a marker for the exam type (histology and cytology) and colored by the exam result. Middle: A histogram of the time between visits. Right: The proportion of female states (normal in blue, low-grade in orange and high-grade in red) in three age intervals.

The NCCSP data is strongly skewed towards disease-free cases with more than 85 % of the individual results being normal and fewer than 5 % high-grade results. Due to the screening recommendations not being strictly adhered to in practice, the histories are irregular in time. This irregularity poses a significant challenge in prediction tasks if the time between the last examination and the time to predict amounts to several years (e.g. > 4 years). The panel in Fig. 1, illustrates these characteristics of the NCCSP data by showing to the left a Lexis diagram depicting screening histories, a histogram of screening intervals in the middle and a histogram of the proportion of female states in three age intervals to the right.

The Lexis diagram in Fig. 1 illustrates the scarcity in screening histories sampled from the NCCSP data, where the median number of exams is 6. The histogram of screening intervals shows that the time between exams varies from just over 1 month and up to almost 20 years, illustrating data irregularity. Finally, the proportion of states is changing with female age, containing about 0.87 normal results for females younger than 36 and up to 0.93 normals for 46–69+ year old females. This drift in the state distribution could be attributed to changes in female lifestyle and screening habits.

### State of the art

Popular prediction models in biomedical applications include *logistic regression*^9^ (LR), *random forest*^10^ (RF) and *gradient tree boosting*^11^ (GTB). Ensemble methods such as RF and GTB may be strong performers on imbalanced data^12^ but neither of these models is typically used with time-dependent data. Popular models in time-series prediction tasks such as *long short-term memory*^13^ (LSTM) networks expect regular and sufficiently sampled data. However, this is not the case with the NCCSP data, as described in the previous section.

An alternative to the LSTM, also capable of modelling cervical cancer data, is a continuous-time *hidden Markov model* (HMM) developed in a recent study^14^ for the disease dynamics observed in cervical cancer screening data. The model was learned from a subset of NCCSP data and validated against a hold-out set by using the HMM as a stochastic simulator to derive Kaplan-Meier estimates. However, the study did not evaluate the HMM on risk prediction tasks or presented a method for generating such predictions from the model.

A later work^15^ introduced a *matrix factorization* (MF) framework using historical data for cervical cancer risk prediction along with a method for classifying the female state from the risk estimate. The MF has become a popular approach to dealing with scarce and irregular data. The proposed framework was compared to a *geometric deep learning* (GDL) model based on^16^, and validated by the *probability of agreement*^17^ between Kaplan-Meier estimates derived from model predictions and a hold-out set. Despite highlighting a heavy class imbalance in their data sample, the authors did not evaluate model calibration or state drift – factors that may explain and affect model performance.

In^18^ the GDL approach was further adapted to handle the scarcity in the cervical cancer screening data. The method was evaluated in numerical experiments by predicting the future risk for individual females at a single randomly chosen time point. To extend methods for personalized risk prediction even further, incremental learning may be incorporated, allowing the models to update risk estimate after more data is available in the future.

### Contribution

This is the first paper comparing several different machine learning methods for cervical cancer risk estimation, focusing on methods for incremental learning from longitudinal data, the impact of data imbalance on model estimates and classification with a time-varying state distribution. Specifically, we compare methods based on HMM, MF and GDL, as well as LR, RF and GTB. Our motivation for including the HMM, GDL and MF is that they were developed to handle scarce and irregular data for cervical cancer screening applications, and we further adapt these herein to incremental learning for longitudinal risk estimation. Moreover, to handle problems with both imbalance and temporal changes in the state distribution, we extend the classification method from^15^ to separate classifiers over different female age intervals. To evaluate their ability to predict the next exam results over time, we compare absolute risk curves derived from model predictions and a hold-out set of screening data to assess model calibration against the trend in the time-varying risk.

The rest of this paper is organized as follows. In “Predicting the risk of cervical cancer development” we outline the risk estimators that are based on extensions to HMM, MF and GDL. “Numerical experiments” section describes the numerical experiments and discuss the results on NCCSP data, followed by a conclusion and outline of future work in “Conclusions and future work”.

## Predicting the risk of cervical cancer development

We represent a cervical cancer screening history with data recorded at times $t_0< t_1< \dots < t_j$ as a set of tuples ${\varvec{y}}_{t_j} = \left\{ (t_i, \rho _{t_i}, x_{t_i}) \right\} _{i=0}^{j}$. The history includes time points $t_i$ representing the female age at visit *i* when she was measured with medical exam $\rho _{t_i}$ to be in state $x_{t_i} = s$. The potential female states $s \in S$ are numerically encoded with $s=1$ for normal, $s=2$ for low-grade and $s=3$ for high-grade.

To estimate the individual future risk of cervical cancer, we assume that we know her screening history up to some time $t_j$. The predicted risk at a later time point ${\hat{t}} > t_j$ is expressed as the triple of conditional probabilities $p(x_{{\hat{t}}} = s \big | {\varvec{y}}_{t_j})$, $s = 1,\,2,\,3$.

In the following sections we provide a detailed description of how we extend existing frameworks based on MF, GDL and HMM to incremental learning for longitudinal predictions. For the LR, RF and GTB predictors we use the implementations from publicly available software^19^.

### Matrix factorization

The matrix factorization (MF) risk estimate as we define it herein is based on the *Shifted Weighted Convolutional Matrix Factorization* (SWCMF) from^15^. The SWCMF assumes that the discrete observed states are possibly inaccurate measurements of a continuous *latent state*, evolving slowly with time for each female. The MF risk estimator requires that we derive such latent state profiles from a hold-out set of screening histories before we can use it for predictions.

By organizing all the states $x_{t_i}$ from a screening history according to female age $t_i$ we obtain a scarce longitudinal vector ${\mathbf {z}}$ spanning $T \ge t_j$ years. Here *T* is the maximum female age in the data and the times $t_0 \le t_i \le T$ for when the female state was measured correspond to the observed entries in ${\varvec{z}}$. Combining *N* such state vectors gives a partially observed matrix ${\varvec{Z}}$.

With the SWCMF model from^15^, we estimate a complete matrix $\varvec{{\widehat{M}}} \in {\mathbb {R}}^{N \times T}$ of latent state profiles using the observed states in ${\varvec{Z}}$ and the medical exam type data. Each row $\varvec{{\widehat{M}}}_n$ in $\varvec{{\widehat{M}}}$ corresponds to a continuous latent profile estimated from ${\varvec{Z}}_n$, and these profiles are used to construct the MF risk estimator.

As in^15^, we assume the probability of observing a female in state $x_{t_i} = s$ at time $t_i$ given the latent state $m_{t_i}$ is given by

1 $$p(x_{{t_{i} }} = s|m_{{t_{i} }} ) = C\exp \left( {{{(m_{{t_{i} }} - s)^{2} } \mathord{\left/ {\vphantom {{(m_{{t_{i} }} - s)^{2} } {2\sigma ^{2} }}} \right. \kern-\nulldelimiterspace} {2\sigma ^{2} }}} \right)$$

where $C = C(m_{t_i})$ is a normalizing factor. Here we estimate $\sigma$ using the same MLE procedure as in^15^. To estimate the risk of some female with screening history ${\varvec{y}}_{t_j}$ being in state *s* at time ${\hat{t}} > t_j$, we consider the posterior predictive distribution

2 $$\begin{aligned} p(x_{{\hat{t}}} = s \big | {\varvec{y}}_{t_j})&\propto \int p(x_{{\hat{t}}} = s \big | {\varvec{m}}, {\varvec{y}}_{t_j})p({\varvec{m}} \big | {\varvec{y}}_{t_j} ) \, d{\varvec{m}} \nonumber \\&= \int p(x_{{\hat{t}}} = s \big | {\varvec{m}}) p({\varvec{m}} \big | {\varvec{y}}_{t_j} ) \, d{\varvec{m}} . \end{aligned}$$

In (2) we assume $y_{{\hat{t}}}$ is conditionally independent from $y_{t_0}, \dots , y_{t_j}$ so the probability of observing state *s* when given history ${\mathbf {y}}$ and latent profile ${\mathbf {m}}$ becomes $p(x_{{\hat{t}}} = s \big | {\varvec{m}})$. Using Bayes’ rule $p({\varvec{m}} \big | {\varvec{y}}_{t_j}) \propto p({\varvec{y}}_{t_j} \big | {\varvec{m}})p({\varvec{m}})$ we get

3 $$\begin{aligned} p(x_{{\hat{t}}} = s \big | {\varvec{y}}_{t_j}) \propto \int p(x_{{\hat{t}}} = s \big | {\varvec{m}}) p({\varvec{y}}_{t_j} \big | {\varvec{m}})p({\varvec{m}}) \, d{\varvec{m}} \end{aligned}$$

In (3) the latent risk prior $p({\varvec{m}})$ is unknown so $p({\varvec{m}} \big | {\varvec{y}}_{t_j})$ is really intractable but following the variational approximation approach we may use $\varvec{{\widehat{M}}}$ to approximate $p({\varvec{m}} \big | {\varvec{y}}_{t_j})$. Thus, we can approximate (3) with

4 $$\begin{aligned} {\widehat{p}}(x_{{\hat{t}}} = s \big | {\varvec{y}}_{t_j}) \propto \sum _{n=1}^N p(x_{{\hat{t}}} = s \big | {\widehat{M}}_{n, {\hat{t}}}) {\widehat{p}}({\varvec{y}}_{t_j} \big | \varvec{{\widehat{M}}}_{n}) . \end{aligned}$$

In (4) we compute $p(x_{{\hat{t}}} = s \big | {\widehat{M}}_{n, {\hat{t}}})$ from (1) and the data likelihood as

$$\begin{aligned} {\widehat{p}}({\varvec{y}}_{t_j} \big | \varvec{{\widehat{M}}}_n) = \prod _{i=0}^j C({\widehat{M}}_{n, t_i}) \exp \left(\frac {({\widehat{M}}_{n, t_i} - x_{t_{i}})^2}{2\sigma ^2}\right), \end{aligned}$$

Moreover, assuming data from a visit at time $t_{j + 1} > t_j$ is added to history ${\varvec{y}}_{t_j}$, we can recursively update the data likelihood by

$$\begin{aligned} {\widehat{p}}({\varvec{y}}_{t_{j + 1}} \big | \widehat{{\varvec{M}}}_n ) \propto {\widehat{p}}(\varvec{{\widehat{M}}}_n \big | {\varvec{y}}_{t_j}) C({\widehat{M}}_{n, t_{j + 1}}) \exp \left(\frac {({\widehat{M}}_{n, t_{j + 1}} - x_{t_{j+1}})^2}{2\sigma ^2}\right) \end{aligned}$$

This recursive update was not described in^15^ and allows us to do efficient adaptive learning by re-estimating the risk when more data is available.

### Geometric deep learning

An alternative to using the SWCMF model^15^ for estimating latent state profiles is to use a *geometric deep learning* (GDL) approach based on^16^. We define the GDL risk estimate based on latent profiles derived with GDL and using (4) for risk predictions. To estimate latent profiles, the GDL leverages two similarity graphs where one encode similarities between screening histories and the other represents the temporal dependency of results. When estimating the latent state profiles, GDL use these graphs to determine the structure of the profiles.

In^15^ the authors used a *k*-nearest neighbour (NN) graph linking together similar histories in addition to a sequential graph for the temporal dependencies. In the *k*-NN graph, each node represents a screening history that is connected to *k* other most similar histories, where the similarity between histories is determined by some pre-defined measure. A potential drawback of the *k*-NN graph is that it each node has to have exactly *k* connections – even if one node is quite dissimilar from the others.

In this paper, we follow^18^ in constructing a graph with a variable number of connections for each node. This graph is learned directly from the data under a smoothness constraint where we assume that certain screening histories exhibit strong similarities to each other. The resulting graph will then contain nodes connecting together histories that are alike in the results and time of visits. Moreover, we assume that the risk of cancer development does not change rapidly within a year and use this to construct the second graph for the temporal dependency of results. Using these two graphs with the GDL we obtain latent state profiles that are changing slowly in time and reflect the similarities between screening histories in the data. The GDL approach is similar to the MF estimate except that they use different constraints to characterize the latent state profiles.

### Hidden Markov model

The HMM risk estimate that we compare to the MF and GDL estimates is based on an extension of the HMM described in^14^ with a prediction module. In the HMM, each observed state is taken to originate from a discrete hidden state indicating the latent risk of cervical cancer development. Here we take the observed states to originate from some hidden states, labelled *normal*, *low-risk* and *high-risk* as in^14^. To define the HMM risk estimate, we first consider the probability

$$\begin{aligned} \alpha (h_{t_j}) = p(h_{t_j} \big | {\mathbf {y}}_{t_j}) \end{aligned}$$

of a female being in hidden state $h_{t_j}$ at time $t_j$, conditioned on her screening history ${\mathbf {y}}_{t_j}$. We use this probability estimate to predict the risk at time ${\hat{t}} > t_j$. To compute $\alpha (h_{t_j})$ we initialize

$$\begin{aligned} \alpha (h_{t_0}) = p(h \big | t_0) p(x_{t_0} \big | h_{t_0}, \rho _{t_0}) \end{aligned}$$

Here, $p(h \big | t_0)$ is a prior over the hidden state at the time of the initial exam, and $p(x_{t_0} \big | h_{t_0}, \rho _{t_0})$ is the probability of the observed state conditioned on the medical exam and hidden state. The estimates for $p(h \big | t_0)$ and $p(x_{t_0} \big | h_{t_0}, \rho _{t_0})$ are available from the parameters of the HMM in^14^. To reach $\alpha (h_{t_i})$ for $t_i > t_0$, we use our previous estimate $\alpha (h_{t_{i - 1}})$ to compute the recursion

5 $$\begin{aligned} \alpha (h_{t_i}) = p(x_{t_i} \big | h_{t_i}, \rho _{t_i}) \sum _{h_{t_{i - 1}}} p(h_{t_i} \big | h_{t_{i- 1}}) \alpha (h_{t_{i-1}}) . \end{aligned}$$

The transition probabilities between hidden states $p(h_{t_i} \big | h_{t_{i- 1}})$ are also given by the HMM parameters^14^.

Having used (5) to obtain our estimate for the hidden state probabilities at time $t_j$, we predict the future risk at time ${\hat{t}} > t_j$ by approximating

6 $$\begin{aligned} p(x_{{\hat{t}}} = s \big | {\varvec{y}}_{t_j}) \propto \int _{\rho _{{\hat{t}}}} \int _{h_{{\hat{t}}}} \int _{h_{t_j}} p(x_{{\hat{t}}} = s \big | h_{{\hat{t}}}, \rho _{{\hat{t}}}) p(\rho _{{\hat{t}}} \big | h_{{\hat{t}}}) p(h_{{\hat{t}}}\big | h_{t_j}) \alpha (h_{t_j}) \, dh_{t_j} dh_{{\hat{t}}} d\rho _{{\hat{t}}} . \end{aligned}$$

The probabilities $p(\rho _{{\hat{t}}} \big | h_{{\hat{t}}})$ we derive from the Poisson intensity estimates presented in^14^. To incorporate more data and update the HMM risk estimate, like we do with MF and GDL, we first update the $\alpha$ estimate with (5) and then estimate the risk with (6).

### Predicting the next state

Using any model to predict the risk of some female being in each state $s \in S$ gives a comprehensive overview of her risk. Predicting the exam result by classifying the female state from these risk estimates amounts to a multi-class classification problem. One approach to this task is to select the most probable state

$$\begin{aligned} {\widehat{x}}_{{\hat{t}}} = \arg \max _{s \in S} \, {\widehat{p}}(x_{{\hat{t}}} = s \big | {\mathbf {y}}_t). \end{aligned}$$

However, this method often fails to predict the minority states because data imbalance shifts the risk inference and classification towards normal. We refer to this classification rule as the *default strategy* as it selects the most probable state without considering data imbalance.

An alternative to the default strategy is to consider state-specific probability thresholds $\left\{ \delta _s \in (0, 1) \right\} _{s \in S}$ adapted to the skewed state distribution. To perform multi-class classification using these thresholds, we can construct a classification rule similar to^15^ where for each state we evaluate

7 $$\begin{aligned} {\widehat{p}}(x_{{\hat{t}}} = s \big | {\mathbf {y}}_t) \ge \delta _{s} \quad \implies \quad {\widehat{x}}_{{\hat{t}}} = s. \end{aligned}$$

If condition (7) holds we predict ${\widehat{x}}_{{\hat{t}}} = s$. We first evaluate (7) for *s* being the high-grade state and then the low-grade state. If the condition is not satisfied for either of these states, we predict normal. This means that we prioritize predicting high-grade over low-grade, and low-grade over and normal as we in our application is more tolerant towards false positives than false negatives.

Furthermore, taking $\delta _s = \delta _s(t)$, we can adapt  (7) to the label drift observed in our data by training a separate classifier for different female age intervals. Since the risk of HPV infection peaks in adolescence and early adulthood, and the risk of cervical cancer peaks in middle aged females^7^, we choose three age intervals: 20–35, 36–45 and 46–69+ for our experiments. Moreover, choosing only three age intervals we aim to avoid overfitting as increasing the number of intervals would also increase the risk of overfitting the classifier in each interval. We refer to using (7) with time-dependent thresholds as the *adaptive strategy*.

To derive the probability thresholds $\delta (T_k)$ for each female age interval $T_k$, we maximize the *K*-category *Matthews correlation coefficient* (MCC)^20^. The MCC summarizes the confusion matrix in a single score

$$\begin{aligned} R_K = \frac{n_+ \times n - \sum _{s\in S} {\hat{n}}_s \times n_s}{\sqrt{(n^2 - \sum _{s\in S} {\hat{n}}_s^2) \times (n^2 - \sum _{s \in S} n_s^2)}} \end{aligned}$$

to measure the quality of multi-class classifications. Here *n* is the total number of test samples, $n_+$ is the number of correct classifications, and $n_s$ and ${\hat{n}}_s$ are the number of times where state *s* was the ground truth and was correctly predicted, respectively. Higher $R_K \in [-1, 1]$ means a more accurate classification. The thresholds $\delta (T_j)$ for age interval $T_j$ are obtained by computing

8 $$\begin{aligned} \max _{\delta (T_k) \in (0, 1)^{S}} R_K({\mathbf {x}}, \mathbf {{\widehat{x}}}). \end{aligned}$$

This maximization problem is solved by using the *differential evolution algorithm*^21^.

## Numerical experiments

The research for this study is approved by the South East Norway Regional Committee for Medical and Health Research Ethics (application ID: 11752). The health registry data used in this study does not originate from clinical trails and therefore the ethical committee granted this study with an exception from obtaining informed consent. All the research conducted herein accommodate the relevant guidelines and regulations.

In numerical experiments we study machine learning methods taking the individual screening history as input for cervical cancer risk prediction. The methods are based on: *hidden Markov model*^14^ (HMM), *matrix factorization*^15^ (MF), *geometric deep learning*^16^ (GDL), *logistic regression*^9^ (LR), *random forest*^10^ (RF) and *gradient tree boosting*^11^ (GTB). To predict the individual risk of cervical cancer development we use (6) for the HMM estimate, and (4) for MF and GDL. Although LR, GTB and RF treat each exam result as independent we facilitate adaptive learning by re-fitting the models with additional data, using the current estimate for model parameters as initialization.

As input to the HMM, MF and GDL predictors we provide all the data up to six months prior to the result we want to predict. For MF and GDL, the input data consist of female states and the corresponding time stamps, while the HMM also utilize exam type information. The input features to LR, RF and GTB combine the cumulative counts of each state conditioned on the exam type over time, together with the corresponding time stamps. For LR we used Z-scoring with parameters estimated from a hold-out set to normalize the features. To derive the latent state profiles used by the MF and GDL estimators, we leverage the exam results and the time stamps from the 10K histories sampled for our training set to construct the input matrix. Moreover, data from the training set is also used to fit LR, RF and GTB.

To simulate an environment for doing longitudinal adaptive learning with MF, HMM and GDL, we masked parts of the screening histories in the test set with a moving window. This way we mimic histories growing over time as a female has more exams. We start by revealing only the first 2 results to fit the estimators, and move forward in time to predict the 3rd result. For any history with more than 3 results, we repeatedly update the model by including the previous data point before we move to predict at the next result.

### The impact of data imbalance on risk estimation

After fitting the models, we assess how well their risk estimates are calibrated using the Brier score. This score measures the agreement between the predicted risk ${\widehat{p}}$ and an indicator $o_{n, {\widehat{t}}}(x_{n, {\widehat{t}}} = s)$ for whether the result was actually $x_{n, {\widehat{t}}}=s$. We compute the Brier score over *N* cases as

$$\begin{aligned} B = \frac{1}{N} \sum _{n=1}^N \left( {\widehat{p}}(x_{n, {\widehat{t}}} = s \big | {\mathbf {y}}_n) - o_{n, {\widehat{t}}}(x_{n, {\widehat{t}}} = s) \right) ^2. \end{aligned}$$

In Table 1, we present Brier scores to evaluate the impact of class imbalance on model estimates. The scores were derived from model predictions aggregated over time and stratified by each ground truth state.

**Table 1:** Brier scores stratified by female states.

Model	Normal	Low-grade	High-grade
MF	0.0830	**0.644**	**0.700**
HMM	0.0410	0.680	0.734
GDL	0.0430	0.683	0.863
GTB	**0.0220**	0.780	0.766
LR	0.0240	0.795	0.777
RF	0.0330	0.790	0.793

The prediction models are matrix factorization (MF), hidden Markov model (HMM), geometric deep learning (GDL) gradient tree boosting (GTB), logistic regression (LR), and random forest (RF).

Significant values are in bold.

From Table 1 we see that we have lower Brier scores for normal states and higher scores for low-grade and high-grade states, which indicates that the prediction models are strongly biased towards the normal state. Thus, the model estimates are clearly affected by the skew in the state distribution. The GDL is especially poor at high-grade predictions but improves on low-grade, while MF is the best calibrated on high-grade followed by HMM.

### Probability thresholding for risk classification

**Figure 2:**
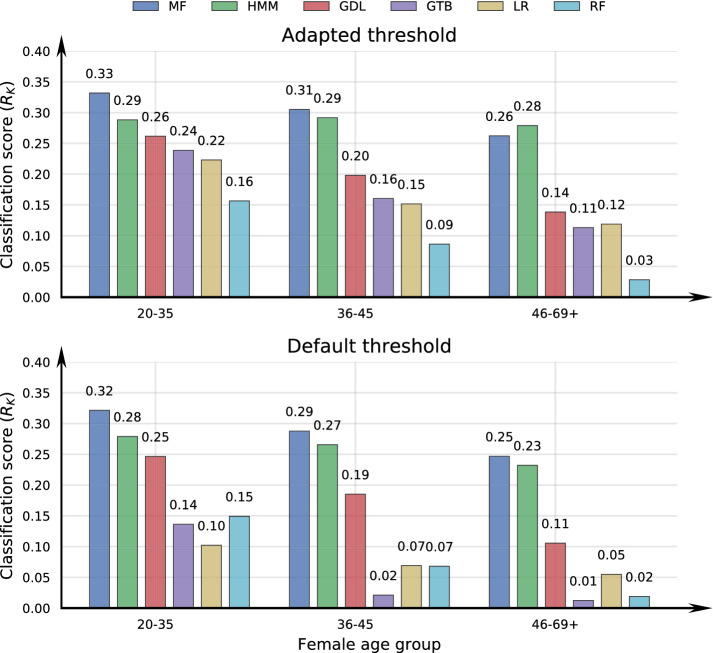
Classification performance as Matthews correlation coefficient ($R_K$) over female age intervals. The prediction models are matrix factorization (MF), hidden Markov model (HMM), geometric deep learning (GDL) gradient tree boosting (GTB), logistic regression (LR), and random forest (RF), combined with either the adapted or default probability threshold method from “Predicting the risk of cervical cancer development”.

One way to alleviate biased probability estimates in classification tasks is to use a classification rule adapted to the data imbalance when converting probabilities into class labels. Using the adaptive thresholding technique from “Predicting the risk of cervical cancer development”, we may also relax the effect of temporal drift in the state distribution by having a different classifier over female age intervals. In Fig. 2 we give the multi-class classification performance as $R_K$ scores achieved with the adaptive and the default classification strategies.

Comparing the $R_K$ scores in Fig. 2 indicates that classification thresholds adjusted to class imbalance improves model performance and is the favourable method over the default strategy, especially with older females. The MF and HMM attains the strongest prediction performance. which is consistent with the model calibration estimates in Table 1, while the GDL, GTB, LR and RF performances decreases more over the age intervals.

### Evaluating classifier performance

**Figure 3:**
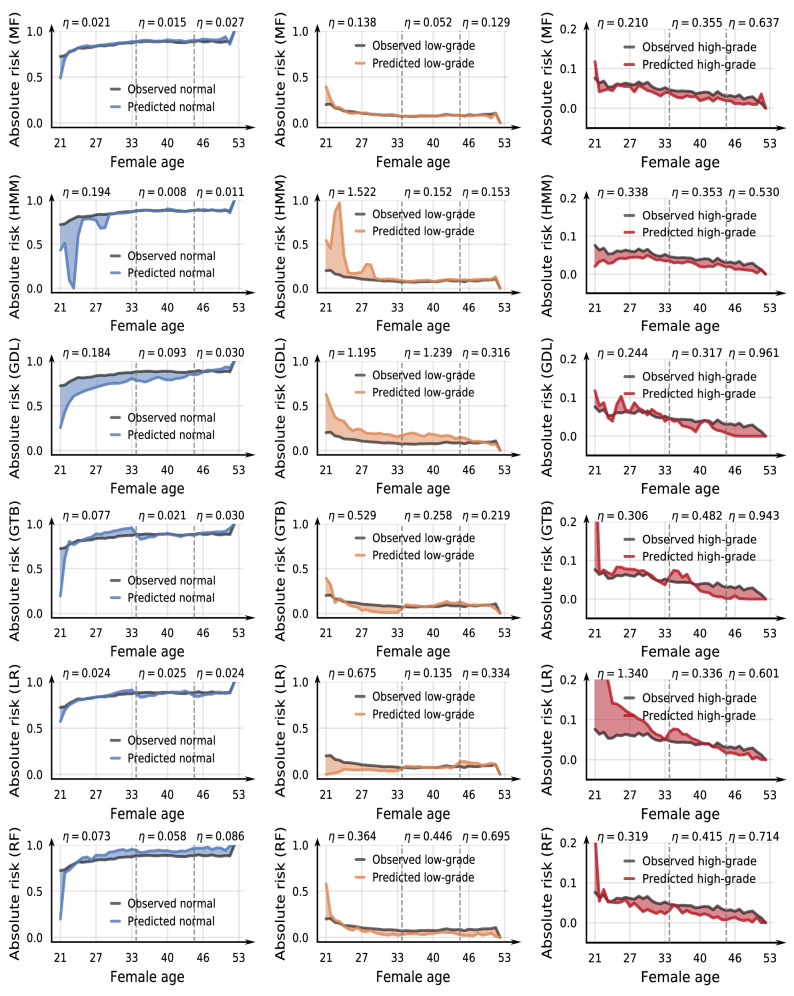
Absolute risk estimated from observed data and model predictions. The $\eta$ score computed with (9) indicates model performance over female age intervals. The prediction models are matrix factorization (MF), hidden Markov model (HMM), geometric deep learning (GDL) gradient tree boosting (GTB), logistic regression (LR), and random forest (RF).

To assess how well the classifiers reflect the trends in the observed data, we compare absolute risk curves from hold-out data and longitudinal model predictions, using the strategy (either default or adaptive probability thresholds) improving on the classification scores in Fig. 2. Here we give absolute risk as the proportion of each state measured over some small time interval of about 10 months. In Fig. 3, we plot risk curves derived from test data and from model predictions. Each row in the panel figure corresponds to a different prediction model and there is one column for each state to more easily distinguish between the curves visually. Stippled vertical lines indicate the age intervals 20–35, 36–45 and 46–69+. Note that the scale on the y-axis differs between the normal/low-grade and high-grade plots to better illustrate the model fit. The colored regions illustrate the difference between the observed (*r*(*t*)) and predicted absolute risk (${\hat{r}}(t)$) at time *t*. To quantify the relative deviation between the absolute risk curves, we define a performance indicator

9 $$\begin{aligned} \eta = \frac{\int \left| r(t) - {\hat{r}}(t) \right| \, dt}{\int r(t) \, dt}. \end{aligned}$$

Ideally, $\eta = 0$, implying perfect classification, while miss-classifications cause the predicted curve to deviate from the test curve, giving $\eta > 0$.

The results in Fig. 3 indicate that the MF model is overall the most accurately calibrated against the trend in the reference curve from the hold-out registry screening data. The predictions from HMM and GDL improve over time, which may be attributed to an increasing amount of training data as older females have typically had more exams. The GTB, RF and LR estimates closely follow the reference curve for normal and low-grade but shows a large deviation in younger females which improves with older females.

## Conclusions and future work

Machine learning methods for more targeted risk stratification can have a high utility to existing cervical cancer screening programs shifting to more personalized screening recommendations. However, deriving such methods from cancer registry data is challenging due to strong class imbalance and a non-stationary data distribution. In this paper, we compare machine learning models based on matrix factorization (MF), hidden Markov model (HMM), geometric deep learning (GDL), logistic regression (LR), random forest (RF) and gradient tree boosting (GTB) in cervical cancer risk estimation, using population-level data from the *Cancer Registry of Norway*.

To define the risk estimators based on HMM, MF and GDL, we extend existing methods with incremental learning mechanisms for longitudinal risk prediction. Results from numerical experiments showed that all the models studied herein suffered from data skewness and were strongly biased towards disease-free results. To predict the individual risk of cancer development we trained separate classifiers adapted to data imbalance over separate female age intervals. Comparing absolute risk curves derived from model predictions and hold-out data showed promising results for matrix factorization to capture the time-varying trend in the observed risk from the data. This methods may thus be useful to improve cervical cancer risk stratification for more personalized screening. We are currently working to elucidate the ability of predictions models to correctly predict individual females using a different representation of model performance.

The methods used in this paper may also be applied to data from other types of mass-screening programs such as breast, colorectal and prostate cancer. In this paper, we focus on using only the routinely collected cervical cancer registry data as we see this to currently have more societal impact and utility for improving healthcare delivery. Expanding the models to include data from more recent screening technology with additional biomarkers and, eventually, individual HPV vaccination status has the potential to improve model performance. In future work we will combine female lifestyle information with registry screening data, believing that including more detailed information about each individual can improve the risk prediction accuracy.

## Data Availability

Due to individual privacy and ethical restrictions, the data used in this study are not publicly available. However, the data can be made available from the Cancer Registry of Norway pursuant the legal requirements mandated by the European GDPR.
